# Surveillance of vector-borne infections: Dogs as sentinel animals for *Orientia* and *Rickettsia* exposure in five regions in Chile

**DOI:** 10.1016/j.onehlt.2026.101437

**Published:** 2026-05-07

**Authors:** Paulina López-Silva, Constanza Martínez-Valdebenito, Ju Jiang, Katia Abarca, Javier López, Allen L. Richards, Thomas Weitzel, Gerardo Acosta-Jamett

**Affiliations:** a Instituto de Medicina Preventiva Veterinaria and Center for Surveillance and Evolution of Infectious Diseases, Facultad de Ciencias Veterinarias, Universidad Austral de Chile, Valdivia, Chile; b Departamento de Enfermedades Infecciosas e Inmunología Pediátricas, Facultad de Medicina, Pontificia Universidad Católica de, Chile; c Naval Medical Research Center, Silver Spring, MD, United States; d The Henry M. Jackson Foundation for the Advancement of Military Medicine, Inc., Bethesda, MD, United States; e Hospital Veterinario Puente Alto, Santiago, Chile; f Allen L. Richards PhD Consulting, Damascus, MD, United States; g Laboratorio Clínico, Clínica Alemana, Facultad de Medicina Clínica Alemana, Universidad del Desarrollo, Santiago, Chile; h Instituto de Ciencias e Innovación en Medicina (ICIM), Facultad de Medicina Clínica Alemana, Universidad del Desarrollo, Santiago, Chile; i Institute of International Health, Charité Center for Global Health, Charité–Universitätsmedizin Berlin, Berlin, Germany; j SENTINET: Surveillance, Epidemiology, and New Technologies for Infectious Emerging Threats, Av. Vicuña Mackenna 4860, Macul, Santiago, Región Metropolitana 7820436, Chile

**Keywords:** Zoonotic diseases, Emerging infectious diseases, One Health, Epidemiology, Prevalence, Scrub typhus

## Abstract

Domestic dogs have been proposed as sentinels for various infectious diseases, including rickettsial infections. In Chile, the only endemic human rickettsiosis is scrub typhus caused by *Candidatus* Orientia chiloensis, an emerging infectious disease, discovered in recent years in southern Chile. The geographic distribution of scrub typhus and other rickettsial infections across Chile is incompletely understood. This study using domestic dogs as sentinel investigated the potential exposure to members of the three main groups of rickettsiae, scrub typhus orientiae (STGO), spotted fever group rickettsiae (SFGR) and typhus group rickettsiae (TGR), in northern, central, and southern Chile. Serological analyses for STGO, SFGR, and TGR were conducted on 1097 dogs from rural and urban areas, which were sampled during 2010 to 2016 in five Chilean regions using a cross-sectional household-based design. Household and dog-level variables were assessed to investigate retrospectively associated risk factors for rickettsial exposure. Overall seroprevalences were 13.8% for STGO, 1.2% for SFGR, and 1.0% for TGR. Higher seropositivity for STGO was associated with dogs from rural areas, male dogs, older dogs, and dog infestation with ticks. Canine seroreactivity to STGO was found across Chile, including regions not known as endemic for scrub typhus. Absence of external deworming was a significant risk factor for SFGR. The present results suggest that during the study period dogs were exposed to rickettsiae over a broader geographic range than previously recognized. Future epidemiological, ecological, and clinical studies are required to confirm the distribution of *Orientia* species and potential endemicity of other rickettsiae in Chile.

## Highlights

• Dogs showed 14% seroprevalence to scrub typhus group orientiae (STGO), suggesting *Orientia* exposure in all study regions.
• Dogs had low levels of seroprevalence to other rickettsial pathogens.
• Rural, male, older, and tick-infested dogs had higher STGO seropositivity rates.
• Dogs are effective sentinels for emerging rickettsial infections.

## 1 Introduction

Domestic dogs live in close contact with humans and share the exposure to most ectoparasites, making them, under the “One Health” approach, an effective sentinel species for many vector-borne zoonotic diseases [1], [2], [3], [4]. Several surveillance studies on rickettsial diseases such as scrub typhus have shown the usefulness of dogs to gain insight into presence and spatial distribution of these pathogens [5], [6], [7], [8]. Compared to human-based surveillance, which often relies on symptomatic cases and access to healthcare, the use of dogs as sentinel species may provide complementary information on pathogen exposure independent of healthcare-seeking behavior. This is particularly relevant in settings where human cases may be underdiagnosed or geographically restricted. Sampling domestic dogs is often more feasible and logistically less complex than conducting large-scale human serosurveys, especially in remote or resource-limited areas. In addition, their roaming behavior increases exposure to environmental sources of infection. Thus, dogs are more likely than humans to encounter certain pathogens, making them valuable sentinels for pathogen circulation, even before transmission to humans might be noted [1], [2], [3], [4].

Rickettsiaceae are obligate intracellular bacteria transmitted by arthropod vectors. Many *Rickettsia* and *Orientia* species are recognized as important human pathogens with zoonotic potential [9], [10]. Over the past four decades, researchers have documented the emergence and re-emergence of rickettsial infections in various regions, most notably scrub typhus caused by bacteria of the genus *Orientia* [11]. This disease was thought to be restricted to the Asia-Pacific region (“tsutsugamushi triangle”), where it causes more than one million cases each year [11], [12]. However, since 2011, several studies have reported multiple autochthonous cases in Chile [13], [14], [15], [16], and a single case in the Middle East [17]. In addition, serological data and molecular evidence from humans, vectors, and animals suggest a wider distribution of the disease, including Africa [18], [19], [20], [21] and America [22], [23], [24], [25], [26]. Scrub typhus in southern Chile is caused by *Candidatus* Orientia chiloensis [27], which has also been detected in trombiculid mites and rodent tissue in this region [28], [29], [30]. Further zoonotic rickettsial species such as *Rickettsia felis* and *Candidatus* Rickettsia andeanae have been detected in ticks and fleas in some regions of Chile [31], [32], [33], [34], [35]. However, to date neither the spectrum nor the geographical range of these rickettsial pathogens are completely known.

The present study analyzed the seroprevalence of *Orientia* and *Rickettsia* in dogs from urban and rural settings in five Chilean regions, using serum samples from a previous household-based project investigating zoonotic pathogens in dogs and dog owners [25], [36], [37].

## 2 Materials & methods

### 2.1 Study population and design

**Fig. 1:**
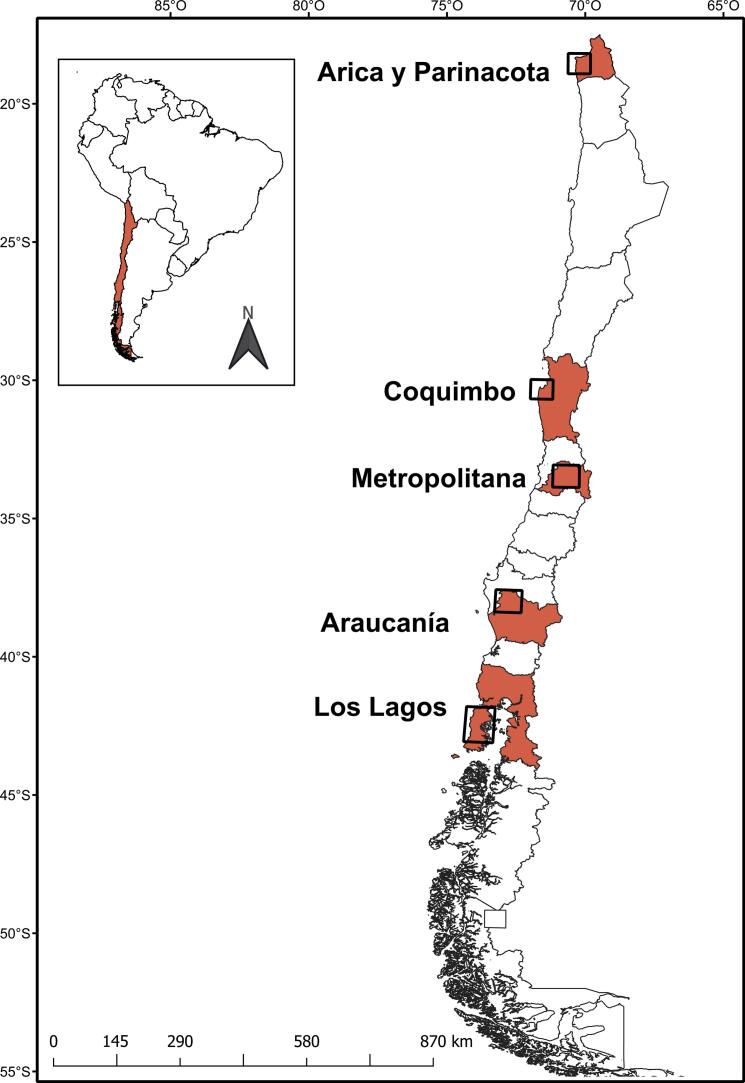
Geographic location of the study area. On the left, Chile (shaded red) is shown within South America. On the right, the specific locations of the study sites within the five regions (in red) of Chile are detailed. (For interpretation of the references to colour in this figure legend, the reader is referred to the web version of this article.)

Five study sites were located in distinct regions in Chile, stretching over a distance of ∼2700 km (Fig. 1). Detailed information on sampling localities and cross-sectional study design including household selection, sample size calculation, sampling techniques, and data collection has been published previously (e.g. [25], [36]). In brief, sample size was calculated per area considering a 10% prevalence, a confidence interval (CI) of 90%, and an error of 4%. Field work was conducted from September 2010 to January 2011 in the Arica y Parinacota Region (18°28′S,70°18′W) and Metropolitan Region (33°37′S,70°34′W), from October 2011 to February 2012 in the Coquimbo Region (29°57′S,71°20′W) and Araucanía Region (37°48′S,72°43′W), and in January 2016 in the Los Lagos Region (41°52′S,73°49′W and 42°28′S,73°46′W). Each site included urban and rural areas. Homes with at least one dog were selected using a double-stratified random sampling by block and household in urban areas and convenience sampling in rural settings. The latter sampling strategy was chosen due to field constraints, including limited accessibility, low population density, and the absence of inhabitants at the time of visits in rural locations.

The survey included a questionnaire on demographics of included dogs and potential factors associated with exposure to zoonotic pathogens. Canine whole blood specimens were centrifuged on the same day; serum was separated, aliquoted, and kept at −20 °C until transport to Santiago, where samples were stored at −80 °C.

### 2.2 Laboratory analysis

Anonymized aliquots were shipped on dry ice to the Department of Viral and Rickettsial Diseases at the Naval Medical Research Center (Silver Spring, MD, USA). There, specimens were tested in a blinded manner using in-house enzyme-linked immunosorbent assays (ELISA) to detect IgG antibodies against scrub typhus group orientiae (STGO), spotted fever group rickettsiae (SFGR), and typhus group rickettsiae (TGR). Antigen preparations included *Orientia tsutsugamushi* (Karp, Kato, and Gilliam strains), whole-cell *Rickettsia conorii* (Malish 7 strain), and *Rickettsia typhi* (Wilmington strain), as previously described [38], with the exception that goat anti-dog IgG HRP (KPL, Gaithersburg, MD, USA) was used as a secondary antibody. Each sample was tested in dilutions of 1:100, 1:400, 1:1600, and 1:6400. Specimens with a total neat absorbance ≥1.000 were considered positive and the titer was defined as the inverse of the highest dilution with an OD of ≥0.2 [38]. In addition, the results of STGO from a subset samples (in Los Lagos Region) derived from an earlier study (using identical methodology) reported preliminarily before [8] were included in the present analysis from a comprehensive point of view.

### 2.3 Statistical analysis

To assess household-level factors (owner education, number of people, number of dogs, rearing practices, and location [urban vs. rural]), and dog-level factors (sex, breed, age, antiparasitic treatment, presence of ticks or fleas) associated with seropositivity to STGO, SFGR, and TGR, univariate logistic regression analyses were performed. Variables with a *p*-value <0.25 were retained for inclusion in multivariate logistic regressions [39], similar to previous studies (e.g. [25]). Statistical analyses were performed using R software version 3.4.1 [40].

### 2.4 Ethical approval

The study protocol was reviewed and approved by the Comité Ético Científico (approval no. 12–170) and by the Comité de Bienestar Animal of the Faculty of Medicine (approval no. 12–033), Pontificia Universidad Católica de Chile in Santiago, Chile, by the Naval Medical Research Center, Silver Spring, Maryland, USA (PJT-16-24), and by the respective health authorities of the five study regions.

## 3 Results

The study included a total of 1097 dogs from 1006 households, of which 550 (50.1%) dogs lived in urban and 547 (49.8%) in rural environments. Sample sizes per region ranged from 202 to 236 specimens. Serological results showed an overall seroprevalence of STGO, SFGR, and TGR of 13.8%, 1.2%, and 1.0%, respectively. Canine seroprevalences varied significantly among the studied regions (Table 1). Of the STGO, the highest prevalences were identified in the Arica y Parinacota Region (23.0%) and in Los Lagos Region (20.8%), followed by Coquimbo Region (10.9%), Metropolitan Region (10.6%), and Araucanía Region (5.5%). SFGR prevalence was highest in the Araucanía region (3.0%), while other regions showed values below 1.0%. TGR had the highest prevalence in the Arica y Parinacota Region (2.4%); other regions exhibited values close to or below 1.0%. Seroprevalences values of STGO and SFGR were higher in rural than in urban areas; this difference was more pronounced for STGO (19.2% vs 8.3%). In contrast, TGR showed similar prevalences in urban environments (1.1% vs 1.0%) (Table 1).

**Table 1:** Canine seroprevalence to group-specific *Rickettsia* and *Orientia* species in urban and rural environments of five regions in Chile.

Region/Site	n	Scrub typhus group orientiae (STGO)			Spotted fever group rickettsiae (SFGR)			Typhus group rickettsiae (TGR)		
		Pos	%	90% CI	Pos	%	90% CI	Pos	%	90% CI
Arica y Parinacota	204	47	23.0	18.7–28.3	1	0.5	0.2–2.3	5	2.4	1.3–5.0
Coquimbo	229	25	10.9	8.0–14.8	3	1.3	0.6–3.3	1	0.4	0.1–2.0
Metropolitana	226	24	10.6	7.8–14.5	1	0.4	0.1–2.1	1	0.4	0.2–2.1
Araucanía	236	13	5.5	3.6–8.6	7	3.0	2.0–5.5	4	1.7	0.8–3.8
Los Lagos	202	42	20.8	16.6–26.0	1	0.5	0.2–2.3	0	0.0	0.0–1.5
Urban	550	46	8.3	6.7–10.5	4	1.0	0.3–1.6	6	1.1	0.6–2.1
Rural	547	105	19.2	16.6–22.1	9	1.7	1.0–2.9	5	1.0	0.5–2.0
TOTAL	1097	151	13.8	12.1–15.6	13	1.2	0.8–1.9	11	1.0	0.7–1.7

Pos, positive; 90% CI, confidence interval 90%.

Multivariate analysis of STGO results revealed that dogs from the Arica y Parinacota Region and Los Lagos Region were about 3-times more likely to be seropositive than those from other regions and that rural dogs were 2.5-times more likely to be seropositive than urban dogs. Additionally, male dogs, older age, and tick infestation were associated with higher likelihoods of seropositivity to STGO (Table 2). Multivariate analysis of SFGR and TGR, showed only absence of antiparasitic treatment as a significant risk factor for SFGR (Table 2).

**Table 2:** Multivariable analysis with binomial errors of factors associated with seropositivity to *Orientia* and *Rickettsia* in dogs (*N* = 1097).

Risk factor	OR	90% CI	*p*
*Scrub typhus group(STGO)*			
Region			
Arica y Parinacota	1.00		
Coquimbo	0.34	0.21–0.53	<0.001
Metropolitana	0.31	0.19–0.50	<0.001
Araucanía	0.16	0.09–0.27	<0.001
Los Lagos	1.08	0.65–1.78	0.812
Site			
Urban	1.00		
Rural	2.53	1.83–3.53	<0.001
Sex			
Female	1.00		
Male	1.74	1.24–2.46	0.008
Age			
< 1 year	1.00		
1–8 years	2.44	1.47–4.24	0.005
>8 years	3.00	1.73–5.40	0.001
Ticks			
No	1.00		
Yes	1.79	1.25–2.59	0.008
*Spotted fever group(SFGR)*			
External antiparasitic			
Yes	1.00		
No	3.04	1.17–8.95	0.065

OR, odd ratio; 90% CI, confidence interval 90%.

## 4 Discussion

The use of sentinel animals enables the surveillance of zoonotic infections and facilitates the identification and monitoring of emerging and re-emerging pathogens, making it an important public health tool [41]. Domestic dogs have several benefits as a sentinel species, as they are exposed to their owners' peridomestic environments including vectors of zoonotic agents. Dogs can be identified conveniently by name and owner, are easily accessible for sampling, and can be relocated for follow-up studies if needed [2]. Due to these benefits, dogs have been used for surveillance studies of multiple human vector-borne pathogens, including *Trypanosoma cruzi*, causing Chagas disease [42], *Borrelia burgdorferi*, causing Lyme disease [43], *Yersinia pestis*, causing plague [44], and different species of *Rickettsia* [45], [46] and *Anaplasma* [36].

To date, scrub typhus caused by *Ca.* O. chiloensis has been identified in Chile from the Biobío Region (36°26′S) southwards to Tierra del Fuego (53°18′S), Magallanes Region [14], [15], [47], where it is transmitted by trombiculid mites (e.g. [14]). Up to now, more than 200 confirmed cases have been reported (authors' unpublished data). The present study, including previously published subset of results [8], estimated canine exposure to *Orientia* over a wider geographical range in Chile. Canine seroprevalence was significantly higher in the Los Lagos Region in southern Chile with the highest number of scrub typhus cases in humans (e.g. outbreak in 2023 [16]) than in the Araucanía Region with fewer cases. Importantly, dogs had also relevant STGO seropositivity rates in the arid areas of the Arica y Parinacota Region (extreme north) and Coquimbo Region (north) as well as the Mediterranean climate Metropolitan Region (central Chile), where to date no human cases have been diagnosed. Climate and ecosystems in these regions differ significantly from the known endemic regions in southern Chile. However, it is known from scrub typhus in the Asia-Pacific region that the infection can occur over a wide range of latitudes with a variety of climatic zones, including dryer ecosystems [10]. Canine STGO exposure was significantly higher in dogs from rural than in those from urban settings. This pattern mirrors the distribution of clinical cases, which occur in people living in or visiting rural environments [16], [48]. This is consistent with the ecology of vector mites (chiggers), which are commonly found in forest clearings, riverbanks, and dense vegetation [10]. In Asia-Pacific, chigger mites are usually reported to occur in “mite islands”, which are often associated with secondary vegetation. Similar ecosystems are common in one of the study sites in the Los Lagos Region (Chiloé Island), where remnants of the original Valdivian temperate rainforest persist [47]. Although various habitats supporting “mite islands” have been recorded, the patchy nature of distribution may be more pronounced in drier climates, where mites tend to be confined to more humid microhabitats [9]. This could be similar in Chile's arid North and Extreme North, where humans and dogs mostly settle in the vicinity of water sources such as rivers where abundant vegetation often occurs. The association of canine STGO seroprevalence with tick infestation is consistent with the fact that ticks and chigger mites might share equal nature environments. Currently, 25 species of trombiculid mites have been described in Chile, most of which are primarily associated with reptiles [28], [30], [49], [50], [51], [52]. However, *Orientia* species has only been detected in four species of rodent-associated mites in southern Chile [28], [30]. Further studies are required to confirm the presence of potential vectors infected with *Orientia* species throughout Chile.

A higher canine seroprevalence with age, as observed in our study, has been described in other pathogens in Chile [36], [53]. Such increase can be caused by (a) constant force of infection in an endemic area, (b) differential rates of exposure in a population experiencing sporadic outbreaks, (c) an increased disease exposure of older dogs, or (d) a recent epidemic. The higher seroprevalence observed in male dogs is probably associated with their roaming behavior and wider ranges of movement compared to females [54]. Dog owners participating in the present project had a very low seroprevalence rate to *Orientia* species [25]. This most probably reflect on the more intense exposure to mite vectors of dogs compared to humans.

Our data suggests that dogs were exposed to SFGR in all study regions, with seroprevalences varying geographically from 0.4% to 3.0%. It is uncertain which rickettsial species caused this seropositivity. So far, clinical cases of spotted fever group rickettsioses have not been identified in humans or animals in Chile. However, human serological data deriving from the present project showed seroprevalence rates of 1.3% to 8.3% with a similar geographical pattern as in dogs (i.e. highest rates in Araucanía Region and Coquimbo Region) and higher rates in rural areas [25]. Up to now, four possible species of SFGR, all of uncertain clinical relevance, have been reported in arthropods and animals in Chile: *Ca*. R. andeanae, *R. felis*, and two incompletely identified species [31], [32], [34], [35], [55], [56], [57], [58]. Possible ticks affecting canines in Chile include the brown dog tick (*Rhipicephalus sanguineus* species complex), which has a wide geographical distribution (latitude ∼18°S to 40°S) and different *Amblyomma* species [31], [32], [34], [59], [60], [61]. Compared to our data, higher SFGR seroprevalence rates have been reported in domestic dogs in other regions of South America. In Mato Grosso in Brazil, for example, dogs had a seroprevalence of 47.5% [62]; in Rio de Janeiro the rate was 67.5% for *R. rickettsii* and 11% for *R. parkeri* [63]. In Bolivia, canine seroprevalence even reached 68.2% [64]. These findings, however, should be interpreted cautiously, since they were obtained using distinct antigens and methods.

The canine TGR seroprevalence was low. This group of rickettsiae includes *R. prowazekii*, the etiological agent of louse-borne epidemic typhus, and *R. typhi*, which causes murine typhus, a poverty related and widely underdiagnosed illness mainly transmitted by rat fleas [65]. WHO reports from the 1940s to 1960s reported murine typhus as endemic in Chile. Since then, however, no autochthonous cases have been detected by modern diagnostic techniques. This could either reflect on improved living conditions in Chile or an underdiagnosis due to absence of routine diagnostic tests and lack of awareness [66]. The slightly higher seroprevalence in urban environments is compatible with transmission by the rat flea *Xenopsylla cheopis,* a vector commonly associated with urban rodent populations such as *Rattus rattus* and *Rattus norvegicus* [67]. Exposure to *R. prowazekii* is unlikely, since the last human cases in Chile were reported in 1939 [68] and its vector, the body louse, does not affect dogs.

This study has several limitations. First, samples were collected between 2010 and 2016 and therefore may not reflect the current epidemiological situation, although the geographical occurrence of rickettsiae within their complex ecological lifecycles (e.g. “mite islands”) are known for their stability over time [10]. Second, the serological assay used *O. tsutsugamushi* antigens, which could lower the sensitivity to detect distinct *Orientia* species; also, the serological detection of IgG cannot distinguish between past and current infection, nor provide information on transmission dynamics. Third, differences in sampling strategies between urban and rural areas, as well as the samples size and lack of adjustment for household clustering, may introduce bias and affect the precision of estimates. Finally, as dogs are not part of the natural transmission cycle of scrub typhus, their seropositivity should be interpreted as an indirect indicator for presence of a pathogen rather than direct evidence for its transmission to humans.

## 5 Conclusion

This study underscores the usefulness of canine surveillance to investigate the spatial distribution of vector-borne infectious agents. The geographical exposure pattern of the present data suggests that STGO, which are known to cause human scrub typhus cases in southern Chile, may have a broader distribution than currently recognized. Future investigations using contemporary data and complementary techniques should focus on the confirmation of *Orientia* in vectors and the surveillance of human cases. Additional human seroprevalence studies using *Ca.* O. chiloensis antigen are currently underway. The detected canine exposure to SFGR and TGR is alarming, suggesting the existence of emerging or re-emerging rickettsial pathogens in Chile. As for scrub typhus, a multidisciplinary One Health approach, searching for such agents in vectors and animals, is required to understand the full spectrum and clinical relevance of rickettsial infections in Chile and neighboring countries.

## Disclaimers

The views expressed in this article reflect the results of research conducted by the authors and do not necessarily reflect the official policy or position of the Department of the Navy, Department of Defense, or the United States Government. At the time of the investigation, A.L.R. was an employee of the U.S. Government, and this work was prepared as a part of his official duties. Title 17 U.S.C. 105 provides that “copyright protection under this title is not available for any work of the United States Government.” Title 17 U.S.C. 101 defines a U.S. Government work as work prepared by a military service member or employee of the U.S. Government as part of that person's official duties.

## CRediT authorship contribution statement

**Paulina López-Silva:** Writing – review & editing, Writing – original draft, Visualization, Formal analysis. **Constanza Martínez-Valdebenito:** Writing – review & editing, Validation, Funding acquisition, Formal analysis. **Ju Jiang:** Writing – review & editing, Validation, Resources, Formal analysis. **Katia Abarca:** Writing – review & editing, Funding acquisition, Conceptualization. **Javier López:** Writing – review & editing, Funding acquisition, Conceptualization. **Allen L. Richards:** Writing – review & editing, Validation, Supervision, Resources, Formal analysis. **Thomas Weitzel:** Writing – review & editing, Funding acquisition, Conceptualization. **Gerardo Acosta-Jamett:** Writing – review & editing, Writing – original draft, Validation, Supervision, Resources, Funding acquisition, Formal analysis, Conceptualization.

## Funding

The study was funded by the 10.13039/501100002850Fondo Nacional de Desarrollo Científico y Tecnológico (FONDECYT N1100809, N1130817, and N1170810) and the Armed Forces Health Surveillance Branch and its Global Emerging Infections Surveillance and Response (GEIS) Section (funding years 2017–2018, ProMIS ID P0032_18_NM_02). NMRC work unit number A0047. P.L.S. and C.M.V. were partially supported by ANID doctoral fellowships (no. 21241474 and n°. 21230036, respectively).

## Declaration of competing interest

None of the authors has a conflict of interest concerning this manuscript.

## Data Availability

Data will be made available on request.
